# Walter Brendel and the dawn of transplantation research in Germany

**DOI:** 10.3389/frtra.2024.1461399

**Published:** 2024-09-17

**Authors:** Walter G. Land

**Affiliations:** ^1^ German Academy for Transplantation Medicine, Munich, Germany; ^2^ Laboratoire d'excellence (LabEx) TRANSPLANTEX, Faculté de Médecine, Université de Strasbourg, Strasbourg, France

**Keywords:** antilymphoyte globulin, heart transplantation, xenograft rejection, extracorporeal shock wave lithotripsy, injury hypothesis, immunological tolerance to horse gammaglobulin

## Abstract

Walter Brendel was a physiologist who headed the Institut of Experimental Surgery at the University of Munich (LMU) from 1961 until 1989. His legendary career began with the development of an anti-human lymphocyte globulin (ALG) at his Institute during the late 1960s. The initial successful treatment of a small number of patients culminated in the co-treatment of the first successfully heart-transplanted patient in Capetown, South Africa (successful reversal with ALG of an acute allograft rejection). Walter Brendel was a pioneering personality whose work has laid a wide platform for the promotion of interdisciplinarily conducted innovative research programs in various domains of translational science and medicine. Among the many innovative achievements, the most notable are: discovery of involvement of the alternative pathway of complement activation in hyperacute xenograft rejection; induction of immunological tolerance to horse IgG as a means to prevent anaphylactic reactions during ALG therapy; development and clinical implementation of the extracorporeal shock wave lithotripsy for extracorporeal destruction of renal and ureteral calculi. The legacy of Brendel continues with the foundation of the *Walter-Brendel Kolleg für Transplantationsmedizin* (i.e., the German Transplant School for Transplantation Medicine), which has been held annually since 1994.

Walter Brendel (*6 November 1922 in Karlsruhe; †29 August 1989 in Munich) was the son of Elisabeth Brendel, born Sigwart, and Wilhelm Brendel, the director of a trading company. He studied medicine in Heidelberg, where he gained his doctorate in 1948. He then worked from 1950 as a resident and later as a senior resident in Physiology at the W.-G. Kerckhoff Institute of the Max Planck Society in Bad Nauheim, Germany. There he researched the circulation and regulation of body temperature until 1961. In 1959 he habilitated (to a “Privatdozent”) at the University in Giessen and began teaching there. From 1961, he headed the Institute of Experimental Surgery at the Surgical Clinic of the Ludwig-Maximilians-Universität (LMU) in Munich, the foundation of which had been initiated by Rudolf Zenker. In 1965 he became an associate professor and in 1969 a full professor of experimental surgery—the first such chair in Germany. From 1969, he headed his Institute for Surgical Research, which had its own building in the Munich University Clinic (“Klinikum Großhadern”) from 1979. Brendel headed the Institute until the beginning of 1989.

Brendel's legendary career began at the Institute for Experimental Surgery with his decision to develop a horse (initially) anti-dog lymphocyte serum/(subsequently) anti-human lymphocyte globulin, a research program that was conducted by a group led by Rudolf Pichlmayr (1). In 1967, after Pichlmayr moved to the Hannover Medical School in Germany, I joined the group to continue the research program on this promising new immunosuppressant (2). (In fact, I was lucky enough to get a position as a research assistant at Brendel's institute, which wasn't that easy: In addition to the usual documents, Brendel—as was typical for him—asked for two requirements: playing an instrument and practizing sport,—I made it with guitar, soccer, and skiing).

The story of the anti-human lymphocyte globulin production then proceeded rapidly: Drawing on extensive experimental studies on a horse anti-dog lymphocye serum administered to kidney transplanted dogs (3) and in close cooperation between the Brendel Institute and the Behringwerke, Marburg, Germany, a horse anti-human lymphocyte globulin (ALG) was produced for clinical use (Figure 1).

**Figure 1:**
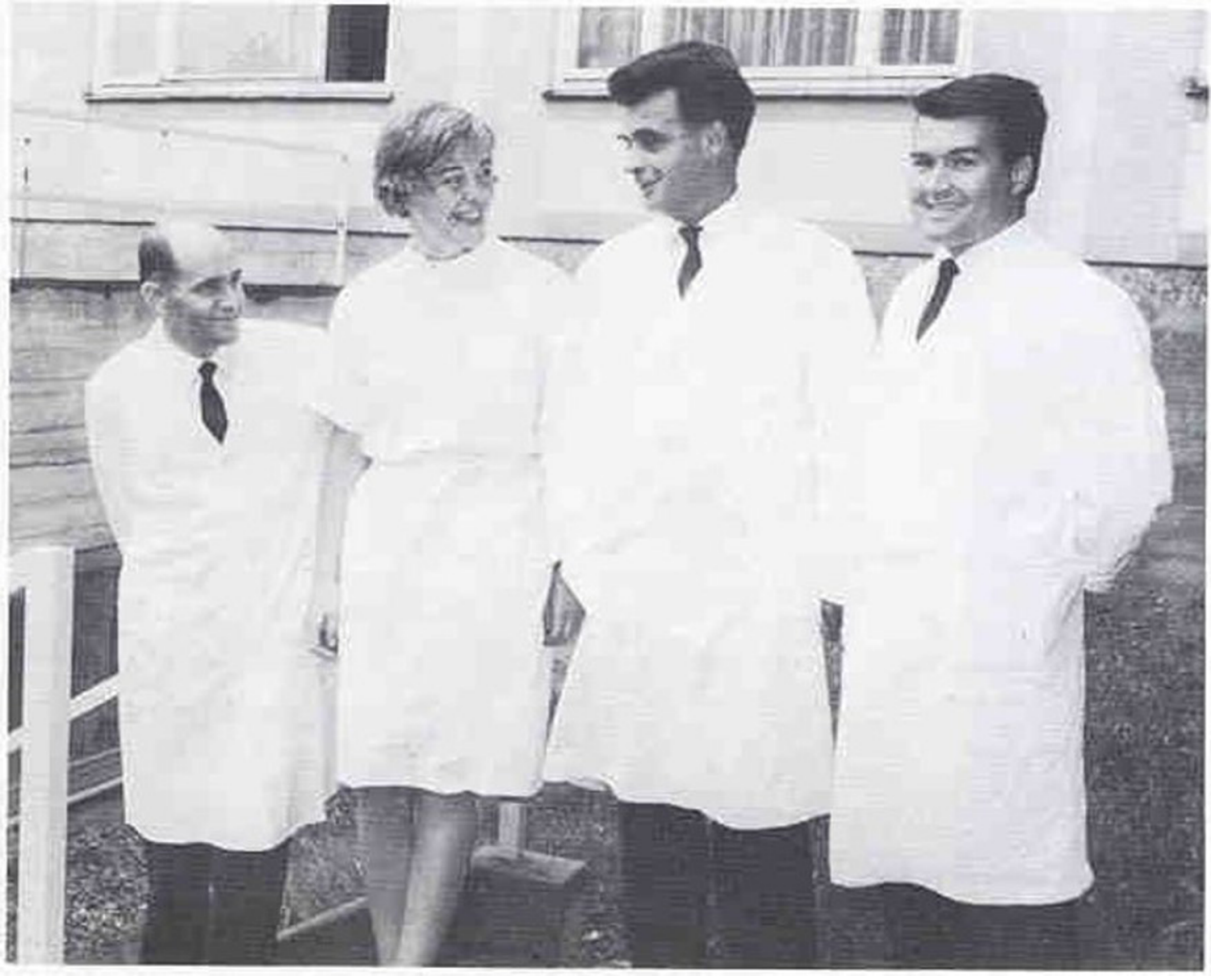
The Munich “ALG-Team“ of the Institute of Experimental Surgery at the University in Munich (LMU), in 1967 (from left to right): Dr. Rudolf Pichlmayr, MTA Christa Schülgen, Prof. Walter Brendel, Dr. Walter Land.

In initial therapy trials on a small series of patients with various autoimmune diseases, ALG was administered intravenously in doses between 1,5 ml and 3, 5 ml daily and found to be effective and safe, notably without causing any anaphylactic adverse effects (4). However, the first transplant patient to receive the Munich ALG was Philip Blaiberg, the first successful heart transplant recipient, operated upon by Christiaan Barnard and his team in Cape Town, South Africa (Figure 2). In June 1968, 6 months post-transplant, the patient experienced a life-threatening acute steroid-resistant rejection episode. Facing the imminent death of his patient, Barnard—recalling Brendel's visit to Capetown in December 1967 at the occasion of the first heart transplant when he told him about the first series of ALG-treated patients with autoimmune disease—decided to administer the Munich ALG as a last resort. Barnard's request for support was swiftly addressed: we promptly dispatched several ampoules of ALG as a “life-and-death shipment” via airmail.

**Figure 2:**
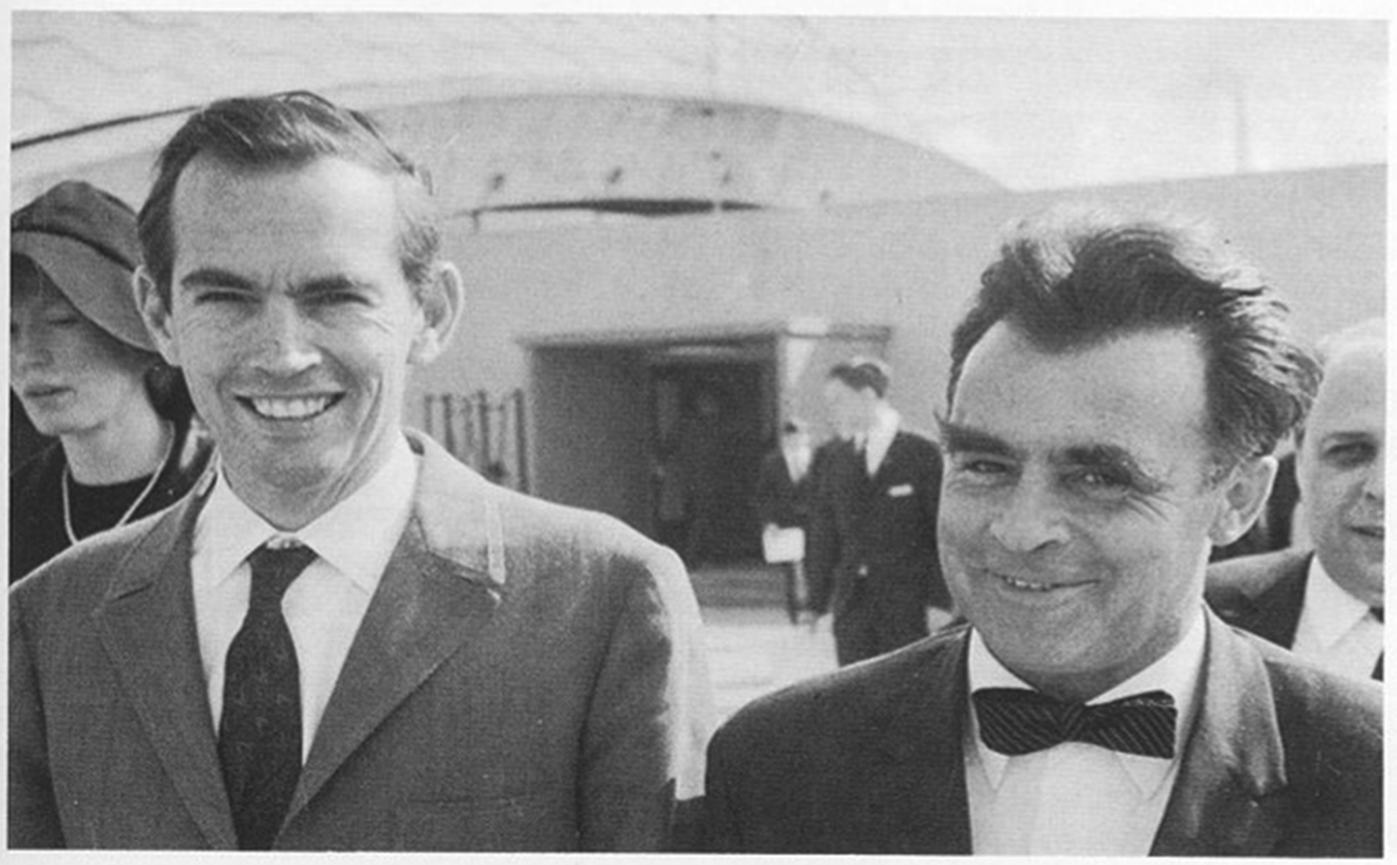
Walter Brendel (right) and Christiaan Barnard on the occasion of the award ceremony for “Jugend forscht”, Frankfurt, Germany, 1969.

Treatment of the patient was initiated immediately, with our guidance provided through daily phone calls. Under daily intravenous administration of 5 ml ALG (plus 2.5 ml twice a week), the acute rejection episode could be completely reversed within three weeks (Figure 3) (5, 6). On Brendel's advice [intuitively estimated, not based on hard experimental nor solid clinical data (!)], ALG was applied intravenously in high doses (7). Undoubtedly, with the successful treatment of an acute cardiac allograft rejection episode in 1968, Brendel and his Institute entered the international stage of experimental and clinical organ transplantation. Later on, the “Munich ALG” was also applied to the first successfully heart-transplanted patient in Germany (8)—this time in the form of an immunosuppressive induction therapy.

**Figure 3:**
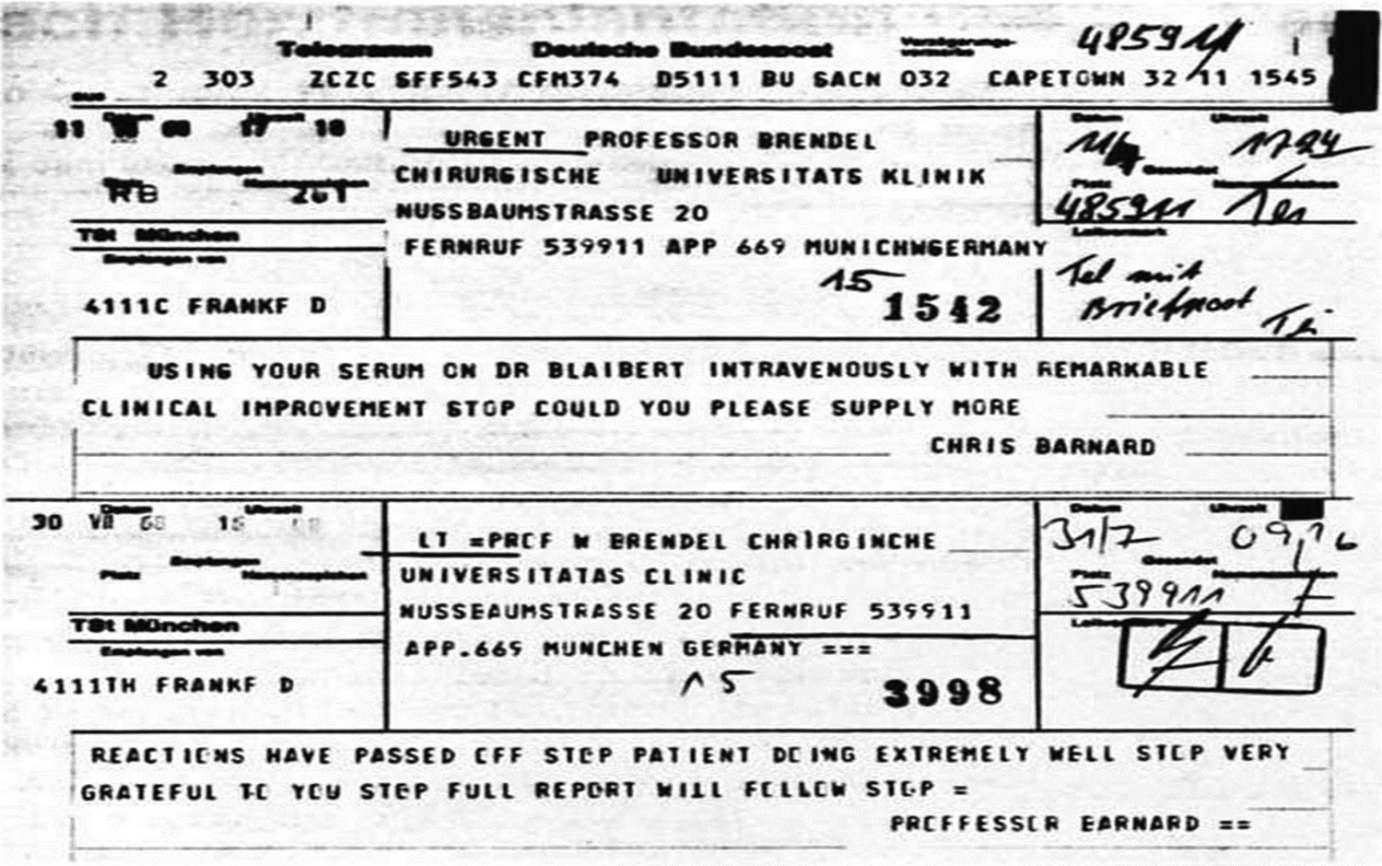
Two telegrams from Barnard to Brendel after the successful treatment of the patient Blaiberg with the “Munich ALG”: partial text: … “using your serum on Dr. Blaiberg intravenously with remarkable clinical improvement—could you please supply more” … “reactions have passed off—patient doing extremely well—very grateful to you—report will follow” ….

Walter Brendel was a pioneering personality whose work at the interface of technology, physiology, and immunology extended far beyond the production of ALG. Freely referencing Alexander von Humboldt, his motto was: “Everything in biology is interconnected”. Accordingly, his work has laid a wide platform for the promotion of interdisciplinarily conducted innovative research programs in various domains of translational science and medicine. His credo was to push boundaries always in search of new horizons, combined with a certain “experimental courage” in the entire field of experimental medicine!

Accordingly, Brendel's ingenuity did not necessarily consist of designing and carrying out elegant experiments, but rather in his ability to spark the creativity of his colleagues to think outside the box and to conceptualize, invent, and devise unusual approaches to tackle a particular scientific challenge. For example, he didn't want an experiment to be carried out that had already been tackled in a similar form elsewhere: His motto was: Innovation first!

To achieve this goal, Brendel sometimes applied unconventional “tactics”. For example, in 1968 at the Second Congress of The Transplantation Society (TTS) in New York, xenotransplantation moved to center stage. Brendel asked me to look for a model experimental set-up that would elucidate the mechanism of hyperacute xenograft rejection. When he realized that I wasn't making any progress, he ordered me to go for a 3-day walk in the forest! After three days, I gained his approval with the idea of setting up an isolated *in vitro* xenohemoperfusion system in which rat kidneys are perfused with dog blood components with constant pressure (9, 10). In these studies, besides others, we were able to show for the first time that the alternative pathway of complement activation plays a dominant role in hyperacute xenograft rejection in widely divergent species combinations. In more detail: In this system, rejection criterion was defined as cessation of xenograft perfusion flow rate with constant perfusion pressure and histologic findings of aggregation of thrombocytes and endothelial lesions. Notably, this *in vitro* experimental set-up allowed for the perfusion of rat kidneys with variously modified dog blood. Evidence for an alternative pathway of complement activation was provided by a series of perfusion experiments using this modification set-up: These experiments showed, among other findings, that hyperacute xenograft rejection did occur when rat kidneys were perfused with complement-containing dog blood that had been depleted of both preformed xenohemoagglutinating antibodies and preformed xenocomplement-fixing antibodies through adsorption (10).

A similar scenario led to the successful induction of immunological tolerance to horse gammaglobulin. Brendel's daily insistence on finding ways to reduce the anaphylactic side effects in ALG-treated patients to horse globulin led to successful attempts to induce immunological tolerance to this xenogenic protein fraction in dogs and humans. By applying Dresser's principle (11), we were able to induce a state of immunological tolerance (as documented by the antigen elimination assay) via intravenous injection of small amounts of highly purified (ultracentrifuged) horse IgG (12, 13).

One of Brendel's main concerns has always been to achieve and maintain a high scientific standard in the research activities tackled at his institute. To this end, he launched two remarkable scenarios, one in the Austrian Alps, the other within the Institute.

1. External event: Since the late 1960s, he organized annual workshops in the winter mountains (which became known as the famous “ski-immunology” Meetings), where he brought his staff together with invited prominent heroes from the fields of immunology, tissue typing, and organ transplantation, who gave “State-of-the-Art” lectures on their recent work. (Notable and renowned attendees of these meetings included prominent leaders, amongst them Sir Peter Medawar, Leslie Brent, Ian Mitchison, Jon van Rood, Ruggero Ceppellini, Roy Calne, Peter Morris, Georges Mathé, Nick Tilney, Tony Monaco, Fritz Bach, David Sachs, and Kathryn Wood). The meetings were characterized by lively discussions, which usually continued into the evening over food and wine, often ending only at midnight.
2. Inside the institute, he encouraged the staff members working on different topics to share their knowledge, experience, concepts, and experimental designs. In fact, Brendel's Institute was known for an outstanding interdisciplinary team spirit that prevailed at this Institute during this time. In order to promote and maintain this interdisciplinary teamwork, Brendel organized social events in which every member of the Institute had to participate, including:
  • the obligate daily coffee break after lunch in a small coffee room where all colleagues sat closely together to discuss the political and academic issues of the day. (Participation in these coffee breaks was a “must” for everybody);
  • an annual dinner in a fish restaurant on the day after carnival (Bavarian “Fasching”);
  • an annual skiing day in the Alps near Munich in winter (Brendel mastered all the slopes with ease, those who didn't dare to tackle a more difficult piste were looked at somewhat “wryly” and with pityingly).

An example of excellent interdisciplinary teamwork involved colleagues working in the field of Hemorrhagic Shock and Resuscitation Regimens, who shared their ideas, knowledge, study designs, and experiments with their colleagues from the field of Transplant Immunology—and vice versa!

The result of this tight-knit interdisciplinary discussion paid off 20 years later when the leader of the shock and resuscitation team, Konrad Messmer, now Director of the Division of Experimental Surgery at the University in Heidelberg asked me—now Head of the Division of Transplant Surgery at the LMU in Munich, Klinikum Grosshadern—to conduct a clinical trial in kidney transplant recipients using the oxygen free radical scavenger superoxide-dismutase (“SOD”) to reduce postischemic allograft reperfusion injury) (14). The clinical trial was successfully implemented and the results obtained (reduced postischemic reperfusion injury → reduction of rejection episodes) formed the basis for the development of the *Injury Hypothesis* (15), which, together with Polly Matzinger's *danger hypothesis* (16), is now regarded as a paradigmatic part of modern immunology.

Of note, the research topics in Brendel's Institute of Experimental Surgery were not restricted to fields of organ transplantation but also included other disciplines such as research on the above-mentioned shock/microcirculation (17) and pathophysiology of traumatic brain edema (headed by Alexander Baethmann) (18).

Amongst these envisaged topics, another highlight of innovative research at Brendel's Institute is the experimental and clinical implementation of the extracorporeal shock wave lithotripsy [ESWL]), a device for extracorporeal destruction of renal and ureteral calculi using shock waves, which was developed in collaboration with Dornier GMBH (19, 20). Originally developed in Munich, this treatment method subsequently spread worldwide and served as a successful conservative therapy for kidney stone disease.

Many of the young researchers at Brendel's Institute went on to have later on stellar careers and leading positions in their disciplines; in the field of organ transplantation in particular Rudolf Pichlmayr and Ulrich Hopt in Germany and Hans-Werner Sollinger in Madison, WI, USA. Additionally, many of today's activities in Germany in the field of experimental/translational medicine, particularly in the area of organ transplantation, can be traced back to the work of Walter Brendel, one of which is based on the aforementioned outstanding interdisciplinary team spirit that prevailed at Brendel's institute.

Indeed, it is this original idea of organ transplantation as a multi-/interdisciplinary speciality that paved the way for the founding of the *Walter-Brendel Kolleg für Transplantationsmedizin* (i.e., the German Transplant School for Transplantation Medicine), which has been held annually since 1994 (21). Accordingly, inspired by Brendel, the idea of unifying the various areas of organ transplantation in a future common new discipline called “*Transplantation Medicine*” was already put forward in the 1980s in an article in which I discussed a subspecialization of colleagues in life (leading) positions who provide special transplantation-related care of the patient (22).

In Germany,—fostered by the continuous activities of the German Academy for Transplantation Medicine over the years (23)—the early visionary proposal culminated recently in its realization in 2023 with the enactment of *The German Transplant Certification* (24)*.* Certified educational programs are offered by the German Transplantation Academy in the form of a 5-day course within the *Walter-Brendel Kolleg für Transplantationsmedizin*, the so-called “*Walter-Brendel Curriculum*” (21), in collaboration with the German Transplantation Society: The legacy of Walter Brendel continues!
